# Plasma Hepatitis E Virus Kinetics in Solid Organ Transplant Patients Receiving Ribavirin

**DOI:** 10.3390/v11070630

**Published:** 2019-07-09

**Authors:** Sebastien Lhomme, Swati DebRoy, Nassim Kamar, Florence Abravanel, David Metsu, Olivier Marion, Chloé Dimeglio, Scott J. Cotler, Jacques Izopet, Harel Dahari

**Affiliations:** 1 National Reference Center for Hepatitis E, Department of Virology, Federative Institute of Biology, CHU Purpan, INSERM U1043, University Toulouse III-Paul Sabatier, 31300 Toulouse, France; 2 The Program for Experimental & Theoretical Modeling, Division of Hepatology, Loyola University Medical Center, Maywood, IL 60153, USA; 3 Department of Mathematics and Computational Science, University of South Carolina-Beaufort, Bluffton, SC 29909, USA; 4 Department of Nephrology and Organ Transplantation, CHU Rangueil, INSERM U1043, University Toulouse III-Paul Sabatier, 31300 Toulouse, France; 5 Department of Pharmacokinetics and Toxicology, Federative Institute of Biology, CHU Purpan, INTHERES, INRA, ENVT, University Toulouse III-Paul Sabatier, 31300 Toulouse, France

**Keywords:** hepatitis E virus, viral kinetics, ribavirin, chronic infection

## Abstract

Hepatitis E virus (HEV) infection causes chronic hepatitis in solid organ transplant (SOT) recipients. Antiviral therapy consists of three months of ribavirin, although response rates are not optimal. We characterized plasma HEV kinetic patterns in 41 SOT patients during ribavirin therapy. After a median pharmacological delay of three (range: 0–21) days, plasma HEV declined from a median baseline level of 6.12 (3.53–7.45) log copies/mL in four viral kinetic patterns: (i) monophasic (*n* = 18), (ii) biphasic (*n* = 13), (iii) triphasic (*n* = 8), and (iv) flat-partial response (*n* = 2). The mean plasma HEV half-life was estimated to be 2.0 ± 0.96 days. Twenty-five patients (61%) had a sustained virological response (SVR) 24 weeks after completion of therapy. Viral kinetic patterns (i)–(iii) were not associated with baseline characteristics or outcome of therapy. A flat-partial response was associated with treatment failure. All patients with a log concentration decrease of plasma HEV at day seven of >15% from baseline achieved SVR. In conclusion, viral kinetic modeling of plasma HEV under ribavirin therapy showed, for the first time, four distinct kinetic profiles, a median pharmacologic delay of three days, and an estimated HEV half-life of two days. Viral kinetic patterns were not associated with response to therapy, with the exception of a flat-partial response.

## 1. Introduction

Hepatitis E virus (HEV) is a single strand positive-sense RNA virus belonging to the *Hepeviridae* family, which causes an estimated 20 million infections annually including about 3.3 million cases of symptomatic hepatitis [1,2]. Hepatitis E (genotype 1 and 2) was considered an exotic disease, present only in developing countries until 1997 when HEV genotype 3 was identified in Western countries, first in pigs [3] and thereafter in persons who had never travelled to highly endemic areas.

Hepatitis E virus causes a self-limited illness in immunocompetent persons and most infections (>95%) are asymptomatic. However, HEV can cause chronic infection in immunocompromised individuals, including solid organ transplant (SOT) recipients and patients with HIV infection or hematological diseases [4]. Rapidly progressing liver fibrosis and cirrhosis develops in 10% of chronically infected immunosuppressed patients [5,6]. Extra-hepatic manifestations are increasingly reported, especially neurological diseases [7,8]. Emergence of virus–host recombinant variants was described in chronically infected patients [9,10].

Decreasing immunosuppressive therapy, especially drugs targeting T-cells, is considered first-line therapy in SOT patients with chronic HEV, resulting in virus clearance in nearly one-third of patients [11]. Antiviral therapy has been studied in SOT patients who do not clear HEV viremia with reduction of immunosuppression. In a multicenter study, 59 patients were treated with ribavirin monotherapy, and a sustained virologic response (SVR), defined as an undetectable plasma HEV RNA level at least 24 weeks after cessation of treatment, was achieved in 78% of patients [12]. The European Association for the Study of the Liver (EASL) recommended ribavirin monotherapy for a duration of 12 weeks for treatment of chronic HEV in SOT patients [2].

Several preliminary predictors of response to ribavirin therapy were proposed: (i) in two patients with ribavirin treatment failure, the emergence of the 1634R mutation was identified in the polymerase at the time of treatment failure [13]. However, the presence of the 1634R mutation in the HEV polymerase at ribavirin initiation was not associated with ribavirin treatment failure [14]; (ii) a lower lymphocyte count at the initiation of ribavirin was identified in treatment failures in comparison to patients who achieved SVR [12]; (iii) the persistence of HEV RNA in stool at the end of ribavirin therapy, despite the absence of HEV RNA in blood, was associated with HEV relapse after ribavirin cessation [15]; finally, (iv) early kinetics of viral load in blood under therapy showed that a decrease of HEV RNA concentration of at least 0.5 log copies/mL from the pre-treatment level within the first week of ribavirin therapy was associated with SVR [16].

A comprehensive characterization of plasma HEV kinetics under ribavirin therapy has not been performed. We sought to characterize HEV kinetic parameters and patterns during ribavirin monotherapy in SOT patients and to evaluate whether viral kinetic patterns are associated with treatment response.

## 2. Materials and Methods

### 2.1. Patients and Ethical Statement

The study was approved by the institutional review board at Toulouse University Hospital, and all patients gave written informed consent to participate. Data were obtained from 41 SOT patients (26 kidney, 9 liver, 3 heart, 1 pancreas, 1 lung and 1 liver/kidney) who received ribavirin for 12 weeks for chronic HEV infection between October 2009 and December 2015 and had sufficiently frequent blood sampling during treatment for viral kinetic analysis. Twenty-two of the cases were described both in our previous study focused on HEV RNA fecal shedding [15] and in the early viral response study [16]. Thirteen patients were only described in the early viral response study [16]. The remaining six patients were included only in this study. Seven patients who were described in our most recent publication [17] were not included due to lack of frequent sampling.

Persistent, unexplained aminotransferase elevations on protocol laboratory measurements prompted HEV RNA testing in SOT patients at our institution. The duration of hepatitis E viremia prior to treatment was calculated from the time of the first positive HEV RNA level. Immunosuppression was decreased in all patients found to have HEV viremia as previously reported [11]. Patients with persistent HEV infection despite a reduction of immunosuppression were treated with 12 weeks of ribavirin.

### 2.2. Biochemical Measurements

Alanine (ALT) and aspartate (AST) aminotransferase levels were measured at pre-treatment (baseline). The ALT normal physiological range was defined as <40 IU/L in men and <35 IU/L in women (adapted from [18]). The AST normal physiological range was defined as <50 IU/L in men and <35 IU/L in women (adapted from [18]).

### 2.3. Detection of Anti-HEV IgM and IgG

Anti-HEV IgM and IgG were detected with an enzyme immunoassay (Wantai Biologic Pharmacy Enterprise, Beijing, China) according to the manufacturer’s recommendations. World Health Organization (WHO) reference material for anti-HEV IgG was used to determine concentrations of IgG. The limit of detection was 0.25 WHO U/mL.

### 2.4. HEV RNA Measurements

All samples were analyzed in the French National Reference Center. HEV RNA was quantified in plasma samples and detected in stool samples using an accredited (ISO 15189) PCR test for HEV3 [19]. HEV RNA measurements were performed at pre-treatment (baseline), at days 3, 7, 15, and 21, and months 1, 2, 3 during therapy, and at months 1, 3, and 6 after completion of ribavirin treatment. The limit of detection for HEV RNA was 100 copies/mL in plasma and stool. HEV RNA at concentrations <100 copies/mL were designated as detected but not quantifiable (DNQ). An SVR was defined as undetectable HEV RNA in plasma for at least 24 weeks after completion of ribavirin therapy.

### 2.5. Genotype Determination

HEV genotype was determined by sequencing a 348-nucleotide fragment within the ORF2 gene [20] and phylogenetic analysis with HEV reference strains (GenBank) [21].

### 2.6. Determination of G1634R Polymorphism by Direct Nucleotide Sequencing

Testing for the G1634R polymorphism was performed on pre-treatment blood samples by sequencing a 451-nucleotide fragment encompassing the C-terminal region of the HEV polymerase as previously described [14].

### 2.7. Measurement of Ribavirin Levels

Plasma ribavirin levels were measured at days 7 and 15 and months 1 and 2 after initiation of ribavirin therapy using a HPLC-UV system, as previously described [22].

### 2.8. Characterization of Plasma Viral Kinetic Patterns

The viral decline patterns were categorized into four main groups by empirical analysis (as previously done to characterize hepatitis C virus (HCV) kinetics under silibinin treatment [23]). Each identified slope (or phase) was calculated by linear regression. We defined a viral decline as monophasic (i.e., single phase of viral decline) if only one phase was present or two viral decline slopes were identified but differed by less than a factor of 2. A two-phase (or biphasic) HEV RNA decline was defined in cases where the first phase decline was 2-fold larger (or smaller) than the second phase decline rate. A flat-partial response was defined in subjects who had a first phase viral decline followed by an extremely slow (or flat) second phase until the end of treatment (EOT). A triphasic viral decline was similar to a flat-partial response but with resumption of viral decline before EOT.

### 2.9. Statistical Analysis

Statistical analysis was performed with STATA statistical software, release 14 (STATA Corporation, College station, TX, USA). Descriptive statistics were performed using number and frequency for qualitative data and median range for quantitative data. We excluded from the statistical analyses the flat-partial viral kinetic group (of the 4 identified groups) because it included only two patients. Due to the small number of patients with some kinetic patterns, we combined the data into two main groups: the viral kinetic monophasic pattern and the viral kinetic multiphasic pattern (i.e., biphasic and triphasic). Qualitative variables were compared between groups using chi-square tests (or Fisher’s exact tests in the case of small expected numbers). The student’s *t* test was used to compare the distribution of normally distributed continuous variables (or Mann–Whitney’s test when the distribution departed from normality or when homoscedasticity was rejected). The independent variables initially introduced into the linear model were those associated with the viral kinetic patterns in bivariate analysis (*p* value < 0.20). Then, a stepwise backward regression procedure was applied to assess variables that were significantly and independently associated with the pattern (*p* value < 0.05). All reported *p* values were two-sided, and the significance threshold was <0.05.

## 3. Results

### 3.1. Baseline Characteristics

Demographic data are summarized in Table 1. The mean age at ribavirin initiation was 51 ± 14 years. A majority of patients were male (*n* = 29/41, 71%) and 63% (*n* = 26/41) were kidney transplant recipients. The median HEV RNA concentration was 6.12 (range (3.53–7.45)) log_10_ copies/mL. The most frequent sub-genotype was HEV3f (61%). At ribavirin initiation, 13 patients had persistently positive tests for plasma HEV RNA for more than six months and 25 patients had viremia for 3–6 months. Three patients were treated at less than three months of viremia because they were deemed to be at high risk for developing chronic infection.

Ribavirin therapy was initiated at a median dose of 600 (200–1200) mg per day, which was equivalent to 9.7 (2.7–16.3) mg per kilogram of body weight per day. Initial ribavirin doses were adjusted on the basis of the estimated glomerular filtration rate (eGFR), as described previously [24]. Although eGFR did not change appreciably during therapy, the ribavirin dose was decreased in 11 patients (27%) because of anemia and was increased in four other patients (9.5%) during treatment. Overall, median ribavirin doses were 600 (200–1200) mg per day at month 1 and 600 (114–1200) mg per day at the EOT.

### 3.2. Response to Ribavirin Therapy

At EOT, 38/41 (93%) patients had undetectable HEV RNA. One-third (13/38) of patients with an EOT response had a post-treatment relapse and none of the three patients with detectable RNA at EOT achieved SVR. Overall, SVR was observed in 25/41 (61%) SOT patients treated with three months of ribavirin. The median log decrease in viral concentration from baseline to day 7, relative to the RNA concentration at day 0, was greater in patients who achieved SVR (14% (0%–21%)) compared to those without SVR (7% (0%–15%); *p* = 0.03). All 10 patients with a log concentration decrease at day 7 and >15% of the log concentration at day 0 achieved SVR. The positive predictive value (PPV) was 100% and the negative predictive (NPV) value was 50%. The sensitivity was 46% and the specificity was 100%. Nineteen patients had undetectable HEV RNA in stool at EOT, 89% of who achieved SVR.

### 3.3. Viral Kinetics

**Figure 1:**
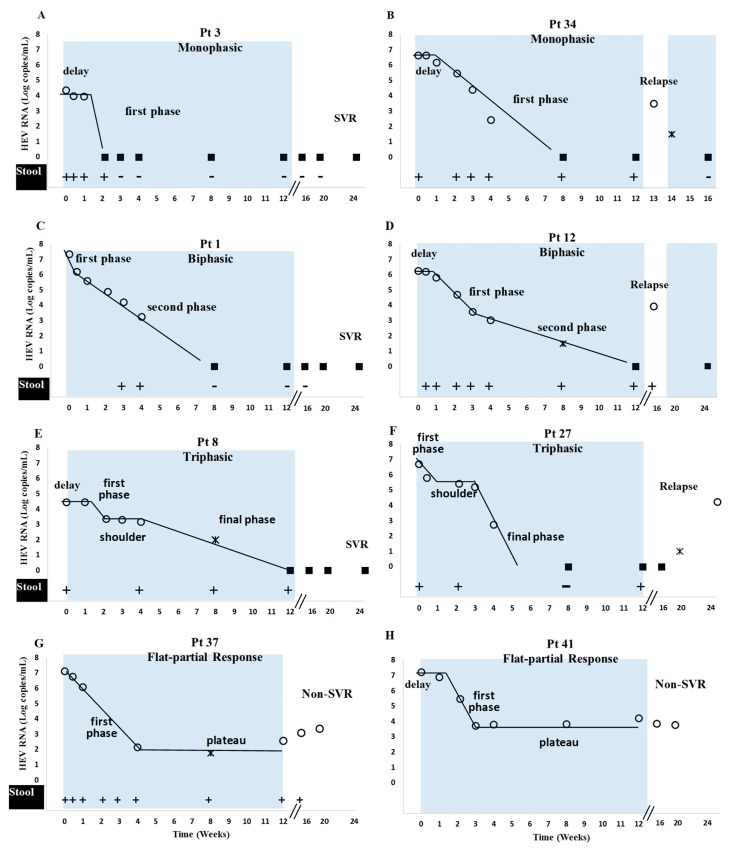
Four identified plasma HEV RNA kinetic patterns during ribavirin monotherapy. Eight representative subjects are plotted. Blue rectangles represent time of ribavirin therapy. Four viral kinetic patterns were identified: (**A**,**B**) monophasic decline, (**C**,**D**) biphasic decline, (**E**,**F**) triphasic decline and (**G**,**H**) flat-partial response. Circles = quantifiable HEV RNA; squares = undetectable HEV RNA; stars = detectable but non quantifiable HEV RNA; +/− = positive/negative HEV RNA in stool. Solid curves were used to illustrate each identified kinetic phase. SVR = sustained virological response. Patients (Pt) 12 and 34 were retreated with ribavirin for 24 weeks and achieved SVR (not shown).

After a short median delay t_0_ = 3 (0–21) days, during which viral load remained at the pre-treatment level, HEV declined in four main viral kinetic patterns: (i) monophasic (*n* = 18), where the slope of decline of VL remained constant during treatment (median t_1/2_ = 2.2 days) (Table 2 and Table S1, Figure 1A,B); (ii) biphasic (*n* = 13), consisting of two distinct slopes of decline (Table 2 and Table S2, Figure 1C,D). Among the biphasic patients, six patients had a rapid–slow pattern (median first phase t_1/2_ = 1.7 days and second phase t_1/2_ = 5.0 days) whereas seven patients had a slow–fast decline (median first phase t_1/2_ = 2.8 days and second phase t_1/2_ = 0.9 days, Table 2 and Table S2); (iii) triphasic (*n* = 8), characterized by an intermediate “shoulder phase” where VL remained constant or declined very slowly (median slope = 0.01 log copies/mL/day, length = 14 days, and VL = 3.23 log copies/mL), between two rapid viral decay phases (median first phase t_1/2_ = 1.7 days; third (or final) phase t_1/2_ = 2.4 days) (Table 2 and Table S3, Figure 1E,F); (iv) flat-partial responders (*n* = 2), with a rapid decline (median first phase t_1/2_ = 1.5 days) followed by a viral plateau until EOT (median 0.01 log copies/mL/day, VL = 3.02 log copies/mL) (Table 2 and Table S4, Figure 1G,H).

The SVR rate was 67% (12/18) in patients with a monophasic decline, 62% (8/13) in those with a biphasic decline, 63% (5/8) in those with a triphasic decline and 0% (0/2) in patients with flat-partial response. None of the profiles was statistically associated with an SVR (*p* = 0.43) or with HEV RNA negative in stool at EOT (*p* = 0.31). Overall, the mean plasma HEV t_1/2_ (calculated from the first phase of viral decline) was estimated at 2.0 ± 0.96 days. The estimated HEV t_1/2_ was similar among the four viral kinetic patterns (*p* = 0.36) and was not associated with treatment outcome (*p* = 0.63) or the ribavirin concentration in blood (ρ = −0.00083, and *p* = 0.97 at day 7; ρ = −0.21, and *p* = 0.2 at month 1). There was no correlation between the dose of ribavirin (9.7 mg/kg/day (2.7–16.3)) and the duration of the delay preceding the viral decline at the initiation of treatment (t_0_ = 3 (0–21) days; ρ = 0.07 and *p* = 0.64). Interestingly, however, non-SVR patients had a longer delay (median t_0_ = 7 (0–21) days) in comparison to patients who achieved SVR (median t_0_ = 3 (0–21) days, *p* = 0.02). Lastly, there was no correlation between the time for HEV RNA to become undetectable in the blood and the treatment outcome (median delay = 52 (15–84) days for both SVR and non-SVR patients; *p* = 0.74).

**Figure 2:**
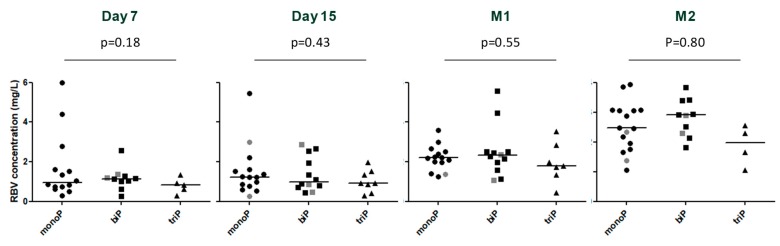
Comparison of ribavirin concentrations and plasma HEV kinetic patterns at days 7 and 15 and months 1 and 2. monoP = monophasic pattern; biP = biphasic pattern; triP = triphasic pattern; grey symbol = 1634R mutation; M1 = month 1; M2 = month 2. The line represents the median.

**Figure 3:**
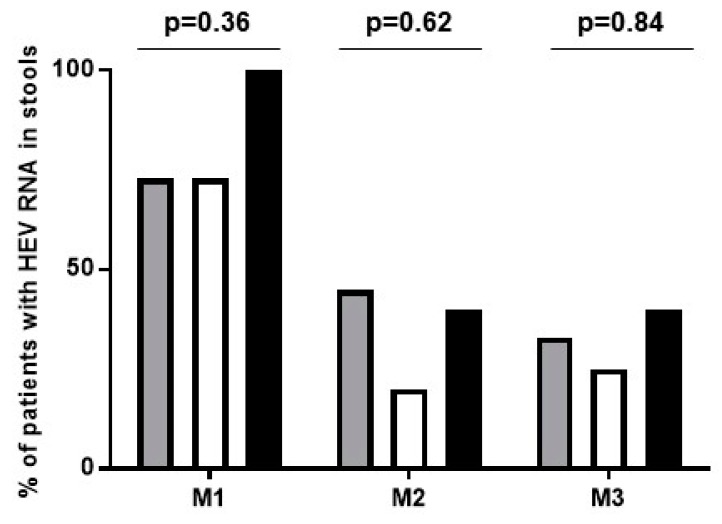
Percentage of patients excreting HEV RNA in stools after 1, 2 or 3 months (M1, M2 and M3, respectively) of ribavirin initiation according to the defined viral kinetic patterns. Grey = monophasic decline; white = biphasic decline; black = triphasic decline. Flat-partial responders (*n* = 2) were excluded (see Figure 1G,H).

Baseline characteristics, including the detection of the 1634R mutation, immunosuppressive drug treatment, decrease in ribavirin dose (44% (8/18) and 31% (4/13) of patients with monophasic and biphasic decline patterns, respectively), ribavirin dose increase (13% (1/8) of triphasic and the two subjects with flat-partial response), and ribavirin trough levels at day 7, day 15, month 1 and at steady state (month 2) (Figure 2) were not associated (all *p* > 0.09) with the four identified viral kinetic patterns. In addition, the ribavirin concentrations were not associated with the decline slopes or the time to TND (all *p* > 0.1). Lastly, there was no association among the viral kinetic patterns and the duration of the HEV RNA fecal shedding at months 1–3 (*p* = 0.36, 0.54 and 0.31 respectively) (Figure 3 and Table S5) (excluding the two patients with flat-partial response in whom HEV shedding in stool was missing in one patient, Figure 1G–H).

## 4. Discussion

Chronic HEV infection was recently recognized as a cause of progressive liver injury and neurologic complications in immunocompromised SOT patients [4] and SVR rates to 12 weeks of ribavirin therapy are suboptimal [12]. Kinetic modeling provides insight into patterns of response to antiviral therapy and viral measurements can be used to optimize treatment of chronic viral infections such as HCV infection [25,26,27]. The current study provides the first detailed viral kinetic assessment of chronic HEV infection during ribavirin therapy. After a median pharmacological delay of three [range = 0–21] days, all patients with chronic HEV treated with ribavirin had a first rapid viral decline in plasma. The estimated half-life of HEV was 2.0 ± 0.96 days, which was not associated with ribavirin concentration in blood. Four viral kinetic patterns were identified: (i) monophasic, (ii) biphasic, (iii) triphasic or (iv) a flat-partial response. Viral kinetic patterns (i), (ii) and (iii) were not associated with outcome of therapy, while the two patients with pattern (iv) failed to achieve SVR. A log plasma HEV concentration decrease at week 1 ≥15% from baseline was associated with SVR, with PPV and NPV of 100% and 50%, respectively, providing a refinement of our previous analysis [15]. Therefore, a day 7 viral load measurement could have predicted SVR with 12 weeks of treatment in 24% of patients in this cohort.

A previous viral kinetic study of HCV showed that a shorter time to HCV target not detected (TND) under pegylated interferon-alpha and ribavirin predicted SVR [28]. In contrast, there was no correlation between the time to HEV TND and SVR. Examination of the HEV RNA decline beyond week 1 showed that 95% of patients (*n* = 39) with viral kinetic patterns (i), (ii) and (iii) reached plasma HEV TND by EOT. Moreover, the two patients who had a flat-partial response did not achieve SVR, suggesting that detectable plasma HEV at EOT is an indication of ribavirin failure. The clinical and demographic characteristics evaluated here were not associated with the viral kinetic patterns. Decrease or increase of ribavirin dose does not seem to influence the kinetic patterns. However, our findings must be confirmed in a more homogenous patient group. While the G1634R mutation in the viral polymerase increases HEV replication in vitro [13], this mutation was not associated with a specific kinetic pattern.

Ribavirin is not a particularly potent antiviral agent and its mechanism of action remains a matter of controversy. The ability of ribavirin monotherapy to eradicate chronic HEV in some immunosuppressed SOT patients is notable. Ribavirin may act to block HEV production since the active metabolite ribavirin monophosphate is a competitive inhibitor of the cellular enzyme inosine monophosphate dehydrogenase (IMPDH), and in vitro experiments suggested that ribavirin activity depends on depletion of intracellular GTP pools, thus impeding RNA replication through inhibition of IMPDH [29]. The strikingly strong effect of ribavirin on HEV is in contrast to the limited/transient effect of ribavirin on HCV [30,31]. If ribavirin acts to inhibit HEV production, the estimated HEV t_1/2_ should reflect the viral t_1/2_ in blood and the magnitude of viral decline from baseline during the first phase may reflect drug efficacy as previously shown during antiviral treatment for hepatitis C, B and D [32,33,34,35]. Interestingly, in vitro studies found that the IMPDH inhibitor mycophenolic acid (MPA) inhibited HEV replication [36,37] and the combination of MPA and ribavirin had a greater impact on HEV replication than MPA or ribavirin alone [36]. However, synergy between ribavirin and MPA was not confirmed in vivo in our previous study [16]. An alternative or complementary explanation for the first phase decline in HEV levels is a rapid turnover of infected cells if ribavirin blocks de novo HEV infection. A strong mutagenic effect of ribavirin on the HEV genome was described in vivo [38], reminiscent of proposed ribavirin effect against HCV [39,40,41,42]. However, using the human liver chimeric UPA/SCID/beige mouse model, Allweiss et al. [43] showed that the administration of ribavirin after the establishment of HEV infection led to a strong reduction of HEV (genotype 1) concentration in both plasma and the liver of infected mice, without evidence of ribavirin-induced cellular damage. Further experiments with humanized mouse models are needed to clarify the mechanism of action of ribavirin in treating HEV genotype 3 [44].

Another obstacle to understanding the mechanism of action of ribavirin against HEV and the viral–host interplay in humans is the evidence for widespread extrahepatic replication of HEV, which is in contrast to chronic hepatitis B, C and D infections that are considered hepatotropic [45]. Extrahepatic HEV replication was described in pigs [46] and rabbits [47,48]. In humans, the detection of HEV particles in urine [49,50] and in stool raises the question of the contribution of the kidney and intestine compartments to the viral kinetic patterns observed here. The presence or absence of HEV RNA in stool at the completion of 12 weeks of ribavirin therapy was previously shown to be a robust predictor of response [15,17]. HEV plasma viral kinetic patterns were not associated with absence of viral shedding in stool at EOT, reflecting the challenge of modeling the response of pathogens with extra-hepatic reservoirs.

## 5. Conclusions

In conclusion, we identified, for the first time, four kinetic patterns during ribavirin treatment for chronic HEV in SOT patients. The viral half-life was estimated at 2.0 ± 0.96 days and a flat-partial response was associated with treatment failure with 12 weeks of ribavirin. The other three viral kinetic patterns did not predict SVR. A 15% HEV RNA log reduction at day 7 identified a subset of patients who achieved SVR with 12 weeks of treatment. Further modeling studies will be required to understand the relationship between plasma viral kinetics and extrahepatic reservoirs of HEV.

## Figures and Tables

**Table 1:** Demographic and clinical characteristics of the 41 patients.

Variable	Value
Age, years (mean, SEM)	51 ± 14
Sex, Male (%)	29 (70.7)
HEV RNA concentration (log copies/mL)	6.12 [3.53–7.45]
HEV genotype, *n* (%)	
3f	25 (60.9)
3c	12 (29.3)
3e	2 (4.9)
3	1 (2.4)
Not determined	1 (2.4)
Type of organ transplant, *n* (%)	
Kidney	26 (63.4)
Liver	9 (22.0)
Heart	3 (7.3)
Kidney/pancreas	1 (2.4)
Lung	1 (2.4)
Liver/kidney	1 (2.4)
Dose of ribavirin (mg/kg/day)	9.7 [2.7–16.3]
Immunosuppressive therapy at the initiation of ribavirin, *n* (%)	
Calcineurin inhibitors	32 (78.0)
Tacrolimus	31 (75.6)
Cyclosporin	1 (2.4)
Antimetabolite	34 (82.9)
Steroids	35 (85.4)
mTOR inhibitors	11 (26.2)
ALT level (IU/L)	119 [24–1506]
AST level (IU/L)	71 [30–1263]
Serum creatinine at the initiation of ribavirin (µmol/L)	133 [40–360]
Lymphocyte count at the initiation of ribavirin (cells/mm^3^)	1200 [178–6000]
Hemoglobin level at the initiation of ribavirin (g/dL)	12.6 [9.3–15.6]
Platelet count at the initiation of ribavirin (cells/mm^3^)	166 [12–1023]
Positive test for anti-HEV IgG antibodies at the initiation of ribavirin, *n*/total (%)	31/41 (75.6)
Positive test for anti-HEV IgM antibodies at the initiation of ribavirin, *n*/total (%)	37/41 (90.2)
Positive test for HEV RNA at the initiation of ribavirin, *n* (%)	41 (100)
Interval between transplantation and initiation of ribavirin (months)	43 [3–268]
Interval between immunosuppression decrease and initiation of ribavirin (months)	4 [0–26]
Interval between diagnosis of HEV infection and initiation of ribavirin (months)	4 [0–47]

Values are median [range] unless indicated otherwise. HEV = hepatitis E virus; ALT = alanine aminotransferase; AST = aspartate aminotransferase.

**Table 2:** Kinetic parameters of the monophasic, biphasic, triphasic or flat-partial response.

Patients	Baseline HEV/RNA	Delay	First Phase Slope	First Phase Length	First Phase	Second Phase Slope	Second Phase Plateau Slope	Mean VL at Plateau	Shoulder Phase Slope	Length of Plateau	Mean VL at Plateau	Final Phase Slope	Final Phase	Time to First DNQ	Time to First	Time to First TND in Stool (Days)
	[log_10_ copies/mL]	[days]	[log_10_ copies/mL/day]	[days]	t_1/2_ [days]	[log_10_ copies/mL/day]	[log_10_ copies/mL/day]	[log_10_ copies/mL]	[log_10_ copies/mL/day]	[days]	[log_10_ copies/mL]	[log_10_ copies/mL/day]	t_1/2_ [days]	[days]	TND [days]	
Monophasic (*n* = 18)	5.60	3	0.14		2.21									15	56	60
IQR	(1.51)	(7)	(0.13)		(1.35)										(35)	(38)
Biphasic (*n* = 13)	6.45	0.00	0.15	15.00	2.05	0.14							2.10	56	56	45
IQR	(0.95)	(3.00)	(0.1)	(4.50)	(1.34)	(0.3)							(4.12)		(56)	(59)
Triphasic (*n* = 8)	5.39	0	0.18	7	1.71				0.01	14	3.23	0.13	2.43	28	56	60
IQR	(1.19)	(6)	(0.21)	(12)	(1.30)				(0.03)	(6)	(1.34)	(0.24)	(4.06)		(49)	(52)
Flat-partial (*n* = 2)	6.67	3.50	0.20	21	1.50		0.01	3.02								

Data are median (interquartile range (IQR)). DNQ = detected but not quantifiable; TND = target not detected; VL = viral load.

## Supplementary Materials

The following are available online at https://www.mdpi.com/1999-4915/11/7/630/s1, Table S1: Details of Patients with Monophasic Viral Load Decline. Table S2: Details of Patients with Biphasic Viral Decline. Table S3: Details of Patients with Triphasic Viral Decline. Table S4: Details of Patients with Flat-Partial Response. Table S5: Viral Kinetic Patterns and HEV RNA in Plasma and Stool at the End of Ribavirin Treatment (EOT).
