# Supplementary material for: Plasma Hepatitis E Virus Kinetics in Solid Organ Transplant Patients Receiving Ribavirin
Source: Viruses. 2019 Jul 9;11(7):630. doi: 10.3390/v11070630 (PMC6669701; doi:10.3390/v11070630)
Supplement: Supplementary file 1 [file viruses-11-00630-s001.pdf]

Supplementary Data

Table S1. Details of Patients with Monophasic Viral Load Decline.

**Patient**	**Response**	**Baseline HEV RNA [log10 copies/ mL]**	**Delay [days]**	**First phase slope [log10 copies/ mL/day]**	**First phase $t_{1/2}$ [days]**	**Time to first DNQ [days]**	**Time to first TND [days]**	**Time to first TND in stool [days]**
**3 ↓**	SVR	4.35	7	0.49*	0.61		15	30
**6 ↓**	SVR	5.27	3	0.44*	0.68		15+	21
**9**	SVR	6.54	0	0.08	3.56		84	60
**10 ↓**	SVR	3.53	0	0.11*	2.72		56	30
**13**	SVR	5.85	3	0.12	2.54	56	84	90
**14**	SVR	4.34	0	0.12*	2.49	15	56	60
**17**	SVR	3.56	3	0.26*	1.17	14	56	60
**19**	SVR	6.98	0	0.12*	2.45		56+	30
**24**	SVR	5.30	0	0.24*	1.26		21	MD
**26 ↓**	SVR	5.23	3	0.30*	1.01		21	MD
**28**	SVR	5.78	7	0.11*	2.85		56	90
**32 ↓**	SVR	6.41	0	0.20	1.48		28	MD
**5 ↓**	Relapse	6.28	7	0.24*	1.26		15	$
**7 ↓**	Relapse	6.22	7	0.13	2.31		56+	$

**15** ↓	Relapse	5.42	21	0.14*	2.14		56	$
**31**	Relapse	6.83	0	0.10	3.15		84	60
**34**	Relapse	6.64	3	0.16	1.87		56	$
**36**	Relapse	5.12	0	0.13	2.28		56	$
18 patients	Median	5.60	3	0.14	2.21	15	56	60
	(IQR)	(1.51)	(7)	(0.13)	(1.35)		(35)	(38)

DNQ = viral load (VL) detected but not quantifiable, assumed VL = 10 copies/mL for calculations; TND = target not detected, assumed VL = 1 copy/mL for calculations; $t_{1/2}$ = half-life; delay = time until treatment induced viral decline; * = minimal estimates, calculated using viral load recorded as TND or DNQ; $^{+}$ = viral blip after TND; $ = positive at end of treatment (EOT); MD = missing data; ↓ = decrease of ribavirin dose.

Table S2. Details of Patients with Biphasic Viral Decline.

**Patients**	**Response**	**Baseline HEV/RNA** [log_{10} copies/mL]	**Delay** [days]	**First phase slope** [log_{10} copies/mL/day]	**First phase length** [days]	**First phase $t_{1/2}$** [days]	**Second phase slope** [log_{10} copies/mL/day]	**Final phase $t_{1/2}$** [days]	**Time to first DNQ** [days]	**Time to first TND** [days]	**Time to first TND in stool** [days]
**1**	SVR	7.35	0	0.25	7	1.23	0.12	2.59		56	60
**4^**	SVR	7.03	0	0.17	7	1.74	0.73*	0.41		15	30
**29**	SVR	6.66	0	0.14	15	2.10	0.06*	4.76		84	90
**35**	SVR	6.75	7	0.17	14	1.79	0.05*	5.80		84	90
**38^ ↓**	SVR	4.70	CE	0.09[#]	15	3.19	0.55*	0.55		21	MD
**40^**	SVR	6.40	3	0.15	18	1.95	0.38*	0.80	28	84	MD
**11^**	SVR	6.48	0	0.06	15	4.74	0.14	2.10	56	84	MD
**16^ ↓**	SVR	5.54	0	0.11	15	2.75	0.30*	1.01		28	30
**30^ ↓**	Relapse	6.93	0	0.06	15	4.66	0.23	1.32		56	21[!]
**2**	Relapse	5.15	7	0.20	14	1.51	0.06*	5.27		56	60
**12**	Relapse	6.24	3	0.15	18	2.05	0.06	5.19	56	84	$
**23**	Relapse	6.24	0	0.33	3	0.92	0.12	2.44		84	15[$]
**33^ ↓**	Relapse	6.45	0	0.13	15	2.29	0.33*	0.91		28	MD
13	Median	6.45	0.00	0.15	15.00	2.05	0.14	2.10	56	56	45

Patients	(IQR)	(0.95)	(3.00)	(0.1)	(4.50)	(1.34)	(0.3)	(4.12)		(56)	(59)

DNQ = viral load (VL) detected but not quantifiable, assumed VL = 10 copies/mL for calculations; TND = target not detected, assumed VL = 1 copy/mL for calculations; $t_{1/2}$ = half-life; delay = time until treatment induced viral decline; * = minimal estimates, calculated using viral load recorded as TND or DNQ; $^{+}$ = viral blip after TND; ^ = the viral decline pattern in these patients was defined by a slower first phase and a faster second phase, which is opposite to the rest of the biphasic viral decline patterns observed; CE = cannot be estimated due to lack of data points; $^{\#}$ = minimal estimate of first phase slope since delay cannot be estimated; * = denotes that the slopes are minimal estimates, calculated using viral load recorded as TND or DNQ; ! = no data at end of treatment (EOT); $ = positive at EOT; ↓ = decrease of ribavirin dose; MD = missing data.

Table S3. Details of Patients with Triphasic Viral Decline.

**Patient**	**Response**	**Baseline HEV/RNA** [log_{10} copies/mL]	**Delay** [days]	**First phase slope** [log_{10} copies/mL/day]	**First phase length** [days]	**First phase: $t_{1/2}$** [days]	**Shoulder phase slope** [log_{10} copies/mL/day]	**Length of plateau** [days]	**Mean VL at plateau** [log_{10} copies/mL]	**Final phase slope** [log_{10} copies/mL/day]	**$t_{1/2}$ of final phase** [days]	**Time to DNQ** [days]	**Time to TND** [days]	**Time to TND in stool** [days]
**8 ↑**	SVR	4.43	7	0.14	7	2.20	0.01	13	3.27	0.06*	5.35		84	90
**20**	SVR	5.03	0	0.37	3	0.80	0.01	12	4.04	0.22*	1.34	28	56	MD
**21**	SVR	4.13	0	0.16	7	1.87	0.05	14	2.69	0.33*	0.90		28	21
**22**	SVR	5.39	3	0.11	21	2.81	[0.01]	7	3.19	0.12*	2.62		56	MD
**39**	SVR	5.96	7	0.15	14	2.02	0.02	28	2.30	0.07*	4.21		84	60
**18**	Relapse	5.57	0	0.19	15	1.55	0.00	13	2.88	0.05*	6.39		84	MD

**25**	Relapse	5.38	0	0.46	3	0.66	0.01	18	4.09	0.53*	0.57		28	$
**27**	Relapse	5.83	0	0.30	3	1.01	0.03	18	5.47	0.13*	2.24		56	60[$]
8	Median	5.39	0	0.18	7	1.71	0.01	14	3.23	0.13	2.43	28.00	56	60
Patients	(IQR)	(1.19)	(6)	(0.21)	(12)	(1.30)	(0.03)	(6)	(1.34)	(0.24)	(4.06)		(49)	(52)

DNQ = viral load (VL) detected but not quantifiable, assumed VL = 10 copies/mL for calculations; TND = target not detected, assumed VL = 1 copy/mL for calculations; $t_{1/2}$ = half-life; delay = time until treatment induced viral decline; * = minimal estimates, calculated using viral load recorded as TND or DNQ; $^{+}$ = viral blip after TND; [ ] = positive slope, VL increasing; * = denotes that the slopes are minimal estimates, calculated using viral load recorded as TND or DNQ; $ = positive at end of treatment (EOT); ↑ = increase of ribavirin dose; MD = missing data.

Table S4. Details of Patients with Flat-Partial Response.

Patient	Response	Baseline HEV RNA [log_{10} copies/mL]	Delay [days]	First phase slope [log_{10} copies/mL/day]	First phase length [days]	First phase $t_{1/2}$ [days]	Second phase plateau slope [log_{10} copies/mL/day]	Mean VL at plateau [log_{10} copies/mL]	Time to TND in stool [days]
**37 ↑**	Relapse	7.12	0	0.18	28	1.66	0.01	2.15[&]	MD
**41 ↑**	Relapse	6.21	7	0.22	14	1.34	0.01	3.89	$
Patients	Median	6.67	3.50	0.20	21	1.50	0.01	3.02	

$t_{1/2}$ = half-life; delay = time until treatment induced viral decline; VL = viral load; [&] = viral load detectable but not quantifiable (DNQ) at day 56 (assumed VL = 10 copies/mL for calculations); MD = missing data; $ = positive at EOT; TND = target not detected; ↑ = increase of ribavirin dose.

Table S5. Viral Kinetic Patterns and HEV RNA in Plasma and Stool at the End of Ribavirin Treatment (EOT).

HEV RNA at EOT		SVR			Non-SVR			
Plasma	Stool	Monophasic	Biphasic	Triphasic	Monophasic	Biphasic	Triphasic	Flat-partial*
neg	neg	9	5	3	1	1	0	0
neg	pos	0	0	0	4	2	2	0
pos	pos	0	0	0	1	0	0	1

neg = HEV RNA not detected; pos = HEV RNA detected; * = flat-partial responders (*n* = 2) were both HEV RNA positive in plasma at EOT measurement in stool was available only in one patient (Figure 1G–H).
